# New Genetically Determined Markers of the Functional State of the Cardiovascular System

**DOI:** 10.3390/genes14010185

**Published:** 2023-01-10

**Authors:** Elena V. Kondakova, Valeria M. Ilina, Lyubov M. Ermakova, Mikhail I. Krivonosov, Kirill V. Kuchin, Maria V. Vedunova

**Affiliations:** 1 Institute of Biology and Biomedicine, Lobachevsky State University of Nizhny Novgorod, 23 Gagarin ave., 603022 Nizhny Novgorod, Russia; 2 Clinic Hospital Number 38, 22 Chernyshevsky St., 603000 Nizhny Novgorod, Russia

**Keywords:** single nucleotide polymorphism, cardiovascular disease, sphygmography, vascular wall, matrix metalloproteinases, methylenetetrahydrofolate reductase, type 1 collagen, apolipoprotein B

## Abstract

Nowadays, cardiovascular diseases (CVDs) occupy a leading position in population mortality. Since it is known that the development of cardiovascular pathologies is determined mainly by the human genetic burden, an urgent task of primary prevention of CVDs is to assess the contribution of gene polymorphism to the formation of cardiovascular risk. The material for the study was the blood of volunteers aged 21 to 102 years. Polymorphisms were determined by real–time PCR. Multichannel volumetric sphygmography was performed to analyze the functional state of the vascular wall. The study revealed that the *rs5742904* polymorphism of the *ApoB* gene was found to be absent in the studied groups of long-livers and descendants of long-livers. Results indicated that the carriage of the heterozygous variant of the *MMP9* polymorphism is associated with a favorable prognosis for cardiovascular system functioning. A tendency towards an increase in the rate of biological age acceleration among subgroups with AA and GG genotypes of the *MMP9* gene and a negative value of biological age acceleration among heterozygous carriers of this polymorphism allele were found. The conducted studies make it possible to identify new associations of the studied polymorphisms with the functional state of the cardiovascular system, which is of great clinical importance and requires further study.

## 1. Introduction

The introduction of the achievements of modern medicine has led to an increase in life expectancy and the number of aging people in recent decades. At the same time, cardiovascular diseases (CVDs) are the leading causes of disability and mortality worldwide [1,2].

Aging is a natural physiological process characterized by progressive loss of tissue and organ function. More importantly, aging is a major risk factor for cardiovascular disease (CVD), which bears the greatest burden on the elderly population and is the leading cause of death worldwide [3]. Cardiovascular disease is not determined by a single cause or a specific sequence of causes. In this case, these diseases are usually called multifactorial [4].

At present, it has become evident that the development of cardiovascular pathologies is determined mainly by the human genetic burden. In particular, many cardiomyopathies [5], channelopathies [6], aortopathies [7], and complex multifactorial diseases, such as coronary heart disease [8], atherosclerosis [9], or atrial fibrillation [10], are of a genetic nature [11]. This has initiated the development of a new field of science—cardiogenetics [12].

Many age-related cardiovascular and cerebrovascular diseases arise due to vascular dysfunction or are aggravated by functional and structural changes in blood vessels. Therefore, it is important to carefully elucidate the fundamental pathophysiological mechanisms underlying the process of vascular aging. Vascular aging is a gradually developing process, characterized by changes in the properties of the vascular wall—progressive thickening, vascular remodeling, and a decrease in elasticity. All this leads to an increase in the rigidity of the vascular wall [3]. Aortic stiffness has a multifactorial etiology and a polygenic mode of inheritance. This reflects the influence of many genes that influence processes, such as cellular signaling, the cytoskeleton, mechanical regulation of vascular structure, and vascular smooth muscle tone [13].

Information about the unique features of an individual can be obtained by studying the molecular structure of sites with polymorphisms [14]. Single nucleotide polymorphism (SNP) is the replacement of one nucleotide in a DNA molecule in the genome of individuals of the same species or between homologous regions of homologous chromosomes [15]. SNPs arise as a result of point mutations and cause the appearance of proteins with different functional activities. On average, there is one SNP per 300 nucleotides [16]. Since single nucleotide polymorphism can lead to changes in the structure and function of the encoded protein, more and more attention has recently been paid to SNPs as a factor in the development of a particular cardiovascular disease. Of particular interest is the search for specific genotypes that worsen the prognosis of the disease.

The polymorphisms A1298C of methylenetetrahydrofolate reductase (*MTHFR)*, R3500Q of apolipoprotein B (*ApoB)*, Gln279Arg of matrix metalloproteinase-9 (*MMP9)*, Asn357Ser of matrix metalloproteinase-12 (*MMP12)*, and C1997A of collagen type 1 α1 (*COL1A1*) are the least studied with regard to their effects on the functioning of the vascular wall and are of particular interest.

A1298C of the *MTHFR* gene leads to the accumulation of homocysteine; this product is toxic to endothelial cells, increases the oxidation of low-density lipoproteins, and has prothrombotic effects [17]. R3500Q of the *ApoB* gene is the main determinant of low-density lipoprotein (LDL) levels and coronary artery calcification [18]. Gln279Arg of the *MMP9* gene results in an amino acid substitution that can increase the activity of the MMP9 enzyme. This SNP is associated with left ventricular dysfunction in coronary artery disease [19]. Asn357Ser of the *MMP12* gene disrupts the protein chain’s conformation and reduces the enzyme’s functional activity [20]. Nowadays, there is very little information about the effect of this gene variant on the occurrence and development of CVD. The *COL1A1* gene encodes pro- α1 of the type 1 collagen chain [21]. When this gene is mutated, a defective protein is synthesized, which significantly shortens the lifespan of vascular smooth muscle cells, contributing to accelerated vascular aging due to stress-induced hyperactivation of beta-galactosidase [22]. At the moment, the *rs1107946* polymorphism of the *COL1A1* gene is considered in the literature as a risk factor for the development of myopathy [23], muscle stiffness and injury [24], and osteoporosis [25]. There are currently no studies on the effect of *rs1107946* on CVD.

Identifying the main stages and components of the pathogenesis of a particular multifactorial disease and the role of SNPs in it will help identify “unfavorable” SNPs, the presence of which worsens the prognosis of the disease under study [14].

The study aimed to identify the effect of single nucleotide polymorphisms in the genes of interest on the functional state of blood vessels.

## 2. Materials and Methods

The studied group consisted of 304 people residing in the Nizhny Novgorod region. The control group of relatively healthy individuals consisted of 234 people aged 21–83 years (mean age: 46.9 ± 1.6 years). The group of long-livers included 49 people aged 86–102 years (mean age: 91.2 ± 0.6 years). The group of descendants of long-livers included representatives of the 1st, 2nd, and 3rd generations, a total of 21 people aged 22–77 years (mean age: 51.6 ± 3.5 years). When examining a group of relatively healthy individuals, the following conditions were put forward as an input control: the absence of chronic and oncological diseases in the acute stage, as well as the absence of acute respiratory viral infections at the time of biomaterial delivery.

The study was conducted at the Department of General and Medical Genetics of the Federal State Autonomous Educational Institution of the Higher Education National Research Lobachevsky State University of Nizhny Novgorod. The work complies with the ethical principles of conducting medical research involving a human subject (9th revision of the Declaration of Helsinki of the World Medical Association, October 2013). The research protocol was approved at the meeting of the Local Ethics Committee (Minutes No. 1, dated 2 December 2020).

The material for the study was whole venous blood with K3-EDTA taken from the study participants after getting their voluntary informed consent. Genomic DNA was isolated from whole blood cells using the DNA-Extran-1 reagent kit in accordance with the manufacturer’s instructions (Syntol, Russia). DNA concentrations were measured using the Qubit dsDNA BR Assay Kit (TFS, USA). Kits were used to determine polymorphisms C1997A of the *COL1A1* gene (*rs1107946*), Gln279Arg of the *MMP9* gene (*rs17576*), Asn357Thr of the *MMP12* gene (*rs652438*), R3500Q of the *ApoB* gene (*rs5742904*) and A1298C of the *MTHFR* gene (*rs1801131*, Russia) (Syntol, Russia). Information about the studied genes, their products and polymorphisms, is shown in Table 1.

Real-time PCR was performed according to the manufacturer’s instructions on a CFX96 Touch amplifier (Bio-Rad, Hercules, CA, USA). For data analysis, two options for processing the results of the study were used: cycle threshold and allelic discrimination analysis, and the results were considered by comparing the position of the unknown sample relative to the controls. Quality control of genotyping was performed by reanalyzing 10% of the samples.

To analyze the functional state of the vascular wall, multichannel volumetric sphygmography was performed using a BOSO ABI-SYSTEM 100 device (BOSCH + SOHN GmbH u. Co. KG, Jungingen, Germany). To determine the effect of the presence of SNPs on changes in the parameters of the vascular wall and an increase in the risk of CVDs, we used sphygmography data from 247 relatively healthy volunteers (194 females and 53 males) aged 21–101 years who were screened for the SNPs of the studied genes. The following indicators were analyzed during statistical processing: ankle-brachial index (ABI) for the right and left limbs, carotid-femoral pulse wave velocity (cfPWV), diastolic blood pressure (DBP), systolic blood pressure (SBP), pulse arterial pressure (PAP), blood pressure balance, pulse rate, body mass index (BMI); we also detected cardiac rhythm disturbances. At the same time, increased PAP ≥ 60 mmHg, cfPWV > 10 m/s, and decreased ABI < 0.9 were regarded as signs of damage to the vascular wall.

Ankle brachial pressure index (ABI) is the ratio of SBP at the lower leg to SBP at the shoulders. The index reflects the degree of stenosis or occlusion of the lower limb arteries as a result of atherosclerosis; it is a screening test for assessing the state of peripheral arteries in individuals with a high risk of cardiovascular events [26]. "Brachio-ankle" PWV not only correlates well with aortic PWV but also additionally characterizes the state of limb arteries; therefore, it has proved to be an independent predictor of adverse cardiovascular events and mortality [27].

To determine the biological age, the PhenoAge model [28] was used; it takes into account general clinical parameters (total white blood cell (WBC)count, mean corpuscular volume (MCV), percentage of lymphocytes (LYM (%)), red cell distribution width (RDW-CV (%)) and biochemical parameters of blood tests (albumin, glucose, creatinine, alkaline phosphatase, C-reactive protein), as well as chronological age.

Statistical analysis was performed using the Python SciPy v1.8.0, scikit_posthocs v0.6.7 and R language WRS2 v1.1-4, vcd v1.4-10 packages. The significance of differences in the distribution of allele and genotype frequencies between groups was determined by the χ^2^ test. Intergroup differences in indicators were analyzed using the post hoc pairwise χ^2^ test. Resulting p-values were corrected by the Benjamini–Hochberg FDR correction procedure. Differences were considered significant at a corrected *p*-value (*p*) < 0.05. The significance of differences between groups by real-valued parameters was tested by applying Kruskal–Wallis test due to non-normal distributions (tested by Shapiro–Wilk test). Post hoc analysis was performed by Dunn’s test with further two stage FDR correction. Due to non-normal data further adjustment of models on sex was performed by robust ANOVA with an estimator of location based on medians with post hoc comparison by mcp2a function [29]. Adjustment for sex in case of binary variables was performed by a Cochran-Mantel-Haenszel chi-squared test [30].

## 3. Results and Discussion

Today, cardiovascular diseases (CVDs) are the leading cause of mortality in the world. Since the timely determination of risk factors for the development of pathologies of the heart and blood vessels can affect the quality of life, the influence of single nucleotide polymorphisms on indicators characterizing the elasticity of the vascular wall was determined.

Volunteer DNA samples were studied for the presence of *rs1107946, rs17576*, *rs652438*, *rs5742904*, and *rs1801131* polymorphisms using the Real-time PCR. For all groups of volunteers, single nucleotide polymorphisms were screened for five genes: new candidates for cardiorisk genes, namely, the matrix metalloproteinase genes—*MMP9*, *MMP12*, collagen type 1 α1—*COL1A1*, the methylenetetrahydrofolate reductase gene—*MTHFR*, and the gene encoding the apolipoprotein B protein—*ApoB*.

The frequency of alleles and genotypes distribution was analyzed in three groups: long-livers, descendants of long-livers, and relatively healthy volunteers with no descendants of long-livers. The distribution frequency of the genotypes of the studied polymorphisms is presented in Table 2.

The frequency of the G allele of the *rs5742904* polymorphism of the *ApoB* gene was 100% in the studied groups of long-livers and descendants of long-livers, respectively; the frequency of the GG genotype was also 100% in these groups. Genotypes GA and AA were found only in the group of relatively healthy volunteers (4.2% and 0.9%, respectively).

According to the literature data, *rs5742904* of the *ApoB* gene causes defective binding to the low-density lipoprotein receptor and hypercholesterolemia, increasing atherosclerosis risk [31]. Even though there were no significant differences in the occurrence frequency of the selected polymorphisms in different studied groups (*p* > 0.05), the absence of the *rs5742904* polymorphism of the *ApoB* gene in the groups of long-livers and descendants of long-livers is of interest for further study of these groups.

Since the products of the selected genes can be of fundamental importance for the functioning of the vascular wall, we investigated the effect of single nucleotide polymorphisms in the studied genes on changes in the functional state of the vascular wall. 

Detailed results are presented in Supplementary Material (Figures S1.1–S1.7, S2.1–S2.7, S3.1–S3.7 and S4.1–S4.7). The most important and significant results are shown below.

**Figure 1:**
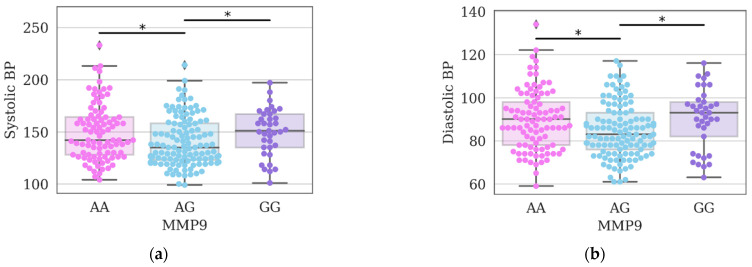
Indicators of systolic (**a**) and diastolic (**b**) blood pressure (BP) in the study group of individuals, depending on the genotypes of polymorphism *rs17576* of the *MMP9* gene; *—differences were considered significant at a corrected *p*-value (*p*) < 0.05 (Kruskal–Wallis test, post-hoc Dunn’s test 2-stage FDR correction).

Although initially it was assumed that the presence of a substitution adversely affects the state of the cardiovascular system (in contrast to wild-type alleles), the analysis of the *MMP9* gene polymorphism revealed a significant increase in systolic and diastolic blood pressure in the group of individuals with the AA genotype compared to the AG genotype (Figure 1a,b).

We performed a sex covariate analysis using Robust ANOVA with median location estimate with mcp2a post hoc comparison. This analysis showed consistent results with previously used methods. We also analyzed the distribution of ages in the study of a number of indicators in the context of the study of *MMP9*. The results of age distributions in various polymorphic variants of the *MMP9* gene are shown in Supplementary Material (Figure S5.1–S5.3). The age distributions in subgroups AA and AG are comparable. An interesting fact is the complete absence of centenarians (over 90 years old) in the subgroup with the GG genotype among all the key features.

**Figure 2:**
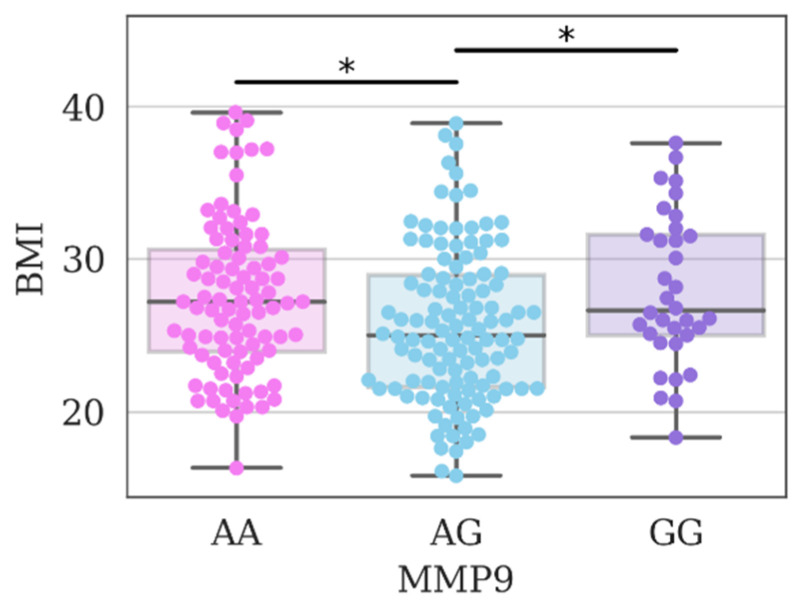
Body mass index values in the study group of individuals depending on the genotypes of polymorphism *rs17576* of the *MMP9* gene; *—differences were considered significant at a corrected *p*-value (*p*) < 0.05 (Kruskal–Wallis test, post-hoc Dunn’s test 2-stage FDR correction).

In addition, in individuals with the genotype AA and GG, the average value of the body mass index is significantly higher than in individuals with the genotype AG, which is another risk factor for the development of cardiovascular diseases (Figure 2).

**Figure 3:**
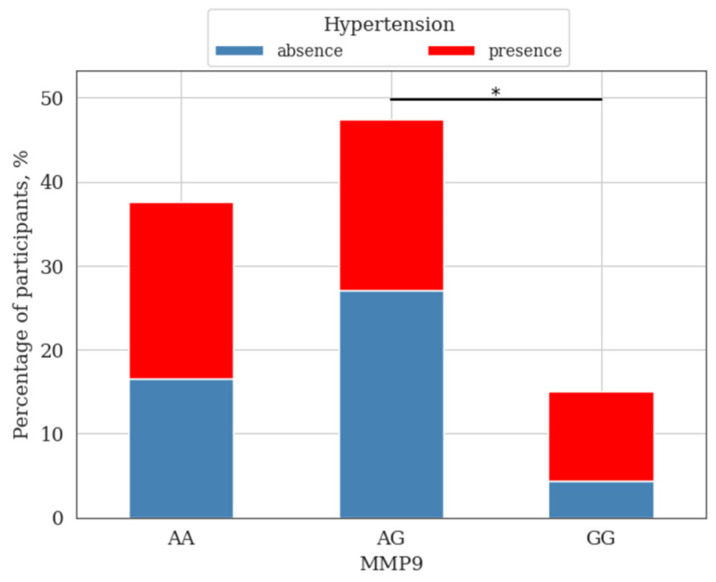
The presence of hypertension in the study group of individuals depending on the genotypes of the polymorphism *rs17576* of the *MMP9* gene; *—differences were considered significant at a corrected *p*-value (*p*) < 0.05 (χ^2^ test, post-hoc pairwise χ^2^ test Benjamini–Hochberg FDR correction).

When assessing the presence of hypertension among the study group of individuals, it was shown that this diagnosis was significantly less common among volunteers with the AG genotype compared to the subgroup homozygous for the minor allele (Figure 3).

When studying other indicators associated with the vascular wall, no significant differences between the genotypes of the *rs17576* polymorphism of the *MMP9* gene were found (Figure S6.1–S6.5).

SNP *rs17576* (Gln279Arg) is a coding variant in exon 6 of *MMP9*, resulting in the replacement of an uncharged amino acid (glutamine) with a positively charged amino acid (arginine), which can increase the enzymatic activity of *MMP9* [19]. This polymorphism probably changes the conformation of the protein, which leads to changes in substrate binding and enzyme activity [32].

The results obtained are consistent with some literature data on the association of the wild-type allele of the *MMP9* polymorphism with pathologies of the cardiovascular system in other populations. In particular, a study conducted on residents of China showed that carriage of the *MMP9 rs17576* AA genotype is associated with a higher risk of symptomatic intracranial atherosclerosis [33]. The microscopic examination of atheromas after carotid endarterectomy revealed a high frequency of carriage of the A allele of the *MMP9* gene (*rs17576*) in the group of patients with unstable atherosclerotic plaques [34]. However, there are some conflicting data about the impact of *rs17576* on cardiovascular system. For example, there is information that the *MMP-9 rs17576* polymorphism is associated with an increased risk of ischemic stroke in the Han Hakka population, and the interaction between *MMP-9 rs17576* and *MMP-12 rs660599* is also associated with an increased risk of ischemic stroke [35]. The contribution of MMPs to vascular aging is further supported by observations of the effect of MMP inhibition on the vessels. It has been shown that tissue inhibitors of MMPs (TIMPs), including four molecules (TIMP-1, -2, -3, -4), reversibly inhibit the proteolytic activity of functional MMPs, and an imbalance of MMPs and TIMPs is associated with arterial hypertension, atherosclerotic plaque formation and aortic aneurysm formation in several experimental models [3].

**Figure 4:**
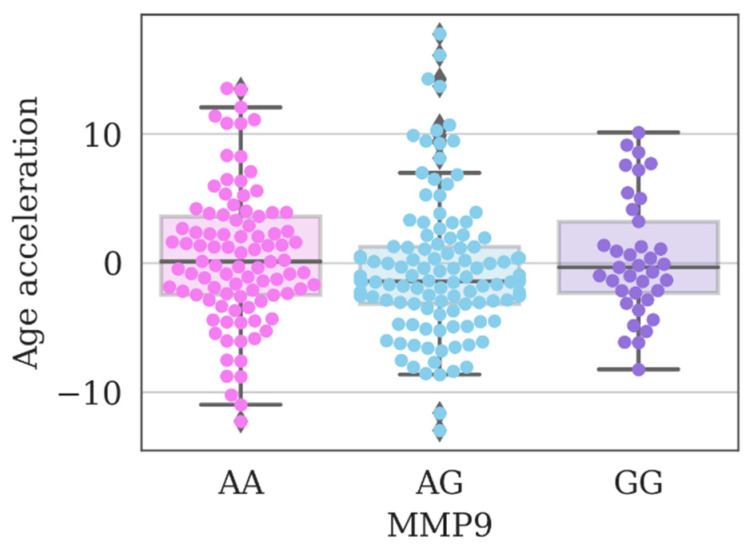
The acceleration value of biological age calculated by the PhenoAge model in the study group of individuals depending on the genotypes of the polymorphism *rs17576* of the *MMP9* gene. No significant differences at a corrected *p*-value (*p*) < 0.05 (Kruskal–Wallis test).

Of particular interest are the results of differences in the age-related acceleration of biological age calculated by the PhenoAge model among individuals with genotype variations of the *rs17576* polymorphism of the *MMP9* gene. The pairwise comparison revealed a significant increase in the acceleration rate among subgroups with AA and GG genotypes and a negative value of biological age acceleration among heterozygous carriers of this polymorphism allele (Figure 4).

At the same time, applying the correction for multiple hypothesis testing, we did not find significant differences between the studied groups. In this case, we can only talk about a tendency of a favorable effect of heterozygous carriage of the allele of the *MMP9* gene polymorphism in comparison with homozygous variants.

Thus, although the AG variant of *MMP9* (*rs17576*) seems to be associated with a favorable state in a number of parameters (lower blood pressure, lower BMI, a tendency to negatively accelerate aging), at the same time, this polymorphic variant is less frequent in the long-livers, which predominantly carry the AA variant. It can be assumed that the AG genotype is critical in terms of vascular aging, which is a reason for further study.

**Figure 5:**
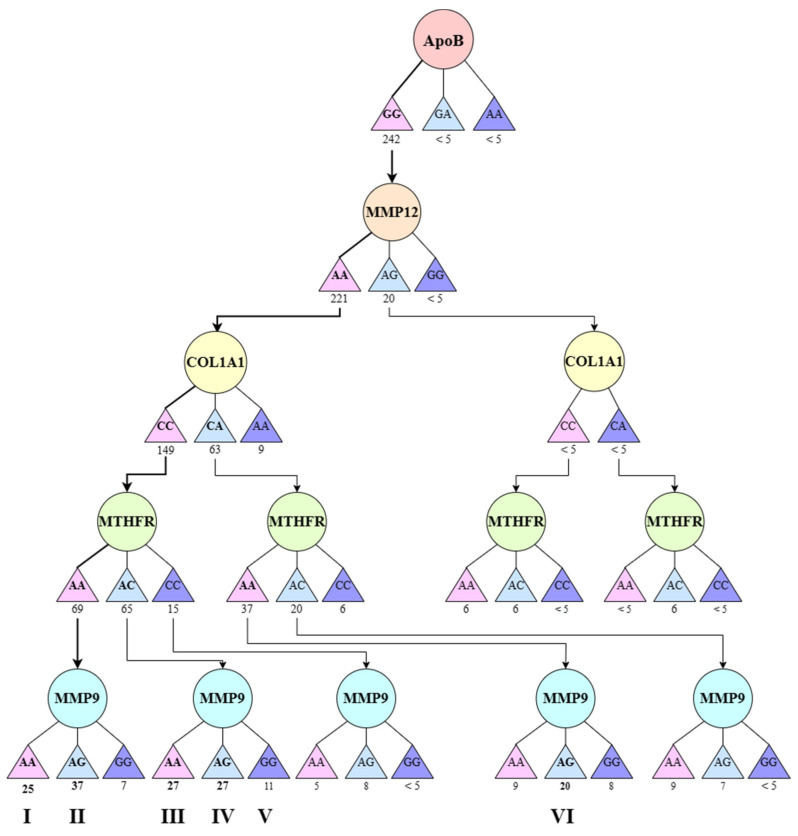
The distribution of study participants by clusters depending on the combination of genotypes in the population of residents of the Nizhny Novgorod region. A cluster was defined as a group of at least 10 participants.

To identify a favorable set of studied polymorphisms, clustering was performed according to the combined distribution of SNPs in the studied genes. As a result, six large clusters (genotype groups I-VI) and a combined group with various combinations of the studied polymorphisms were obtained. The results are presented in Figure 5.

**Figure 6:**
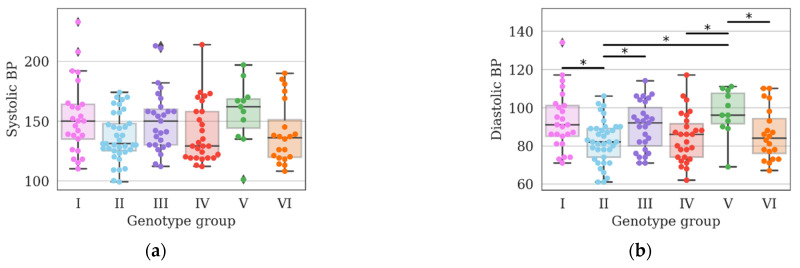
Differences in systolic (**a**) and diastolic (**b**) blood pressure (BP) among the obtained clusters; *—differences were considered significant at a corrected *p*-value (*p*) < 0.05 (Kruskal–Wallis test, post-hoc Dunn’s test 2-stage FDR correction).

Based on the results of the genotype distribution, we studied the previously analyzed parameters within the obtained groups. Systolic blood pressure (SBP) not differ, diastolic blood pressure (DBP) differed between groups I and II, II and III, II and V, IV and V, V and VI (Figure 6a,b). Group V is characterized by the highest blood pressure, both systolic and diastolic.

**Figure 7:**
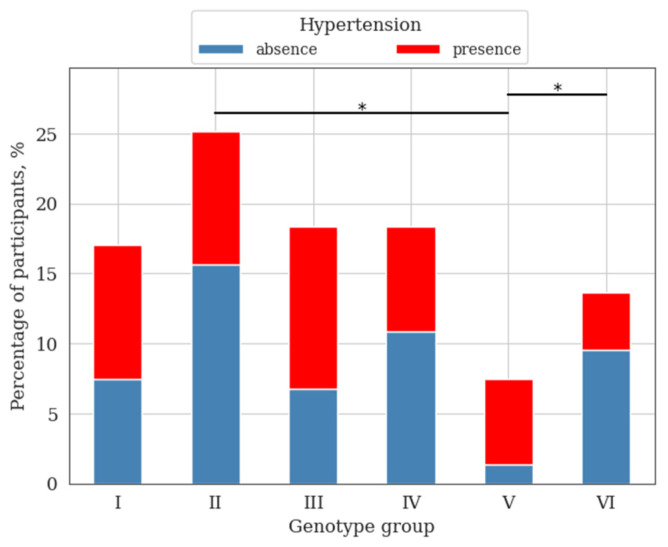
The presence of hypertension among the studied groups, depending on clusters; *—differences were considered significant at a corrected *p*-value (*p*) < 0.05 (χ^2^ test, post-hoc pairwise χ^2^ test Benjamini–Hochberg FDR correction).

Significant differences were found in the as-assessment of the presence of hypertension among groups II and V, V and VI (Figure 7). 

Summing up, group V can be characterized as less favorable in assessing indicators related to the state of the cardiovascular system. The main difference of this group is the GG variant of the polymorphism *rs17576* of the *MMP9* gene.

## 4. Conclusions

The studies revealed that the R3500Q polymorphism of the *ApoB* (apolipoprotein B) gene was absent in the groups of long-livers and descendants of long-livers. This fact is of interest for further research of the *ApoB* gene as a candidate gene in the development of age-associated diseases of the cardiovascular system.

Analysis of the *MMP9* gene polymorphism showed a significant increase in systolic and diastolic blood pressure in the group of individuals with the wild-type genotype compared to heterozygous carriers. It has also been shown that hypertension is significantly less common among volunteers with a heterozygous genotype.

A negative value of biological age acceleration in the PhenoAge model among heterozygous carriers of the *MMP9* polymorphism allele was revealed. The presence of this genotype variant may be associated with a later manifestation of age-related diseases, in particular, cardiovascular pathologies.

Although the AG variant of *MMP9* (*rs17576*) appears to be associated with a favorable state in a number of parameters related to the cardiovascular system, this polymorphic variant is less common in centenarians who predominantly carry the wild variant of the gene (AA). It can be assumed that the AG genotype is critical in terms of vascular aging.

The conducted studies showed conflicting results, while allowing for the identification of new associations of the studied polymorphisms with the functional state of the cardiovascular system, which is of great clinical importance and requires further study.

## Figures and Tables

**Table 1:** The studied genes, their products and polymorphisms.

Gene	Gene Product	Polymorphism
*MMP9*	matrix metalloproteinase-9	*rs17576* Gln279Arg
*MMP12* *COL1A1* *MTHFR* *ApoB*	matrix metalloproteinase-12 pro- α1 chains of type 1 collagen methylenetetrahydrofolate reductase apolipoprotein B	*rs652438* Asn357Ser *rs1107946* C1997A *rs1801131* A1298C *rs5742904* R3500Q Arg3500Gln

**Table 2:** Distribution frequency of *MMP9*, *MMP12*, *COL1A1*, *MTHFR*, *ApoB* gene polymorphisms among the three studied groups.

	Study Groups		
Polymorphism	Long-Livers	Descendants of Long-Livers	Control Group
*rs17576* of the *MMP9* gene	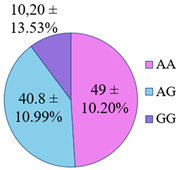	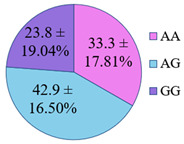	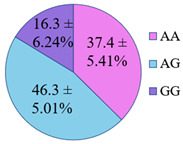
*rs652438* of the *MMP12* gene	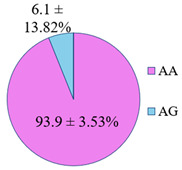	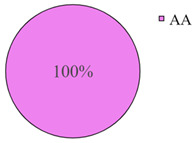	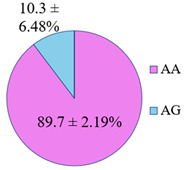
*rs1107946* of the *COL1A1* gene	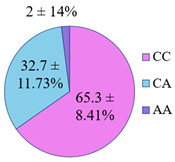	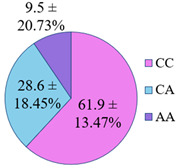	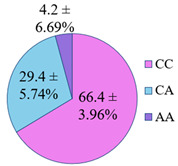
*rs1801131* of the *MTHFR* gene	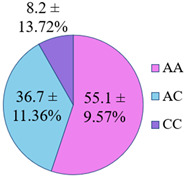	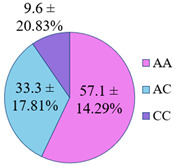	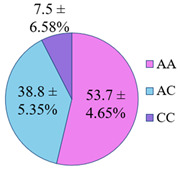
*rs5742904* of the *ApoB* gene	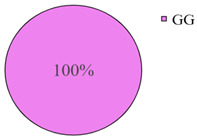	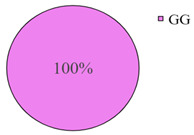	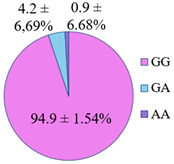

Values are presented as *p* ± *σp%*, where *p* is a percentage, *σp* is a standard deviation of a percentage.

## Data Availability

The original contributions presented in this study are included in the article/Supplementary Material; further inquiries can be directed to the corresponding author.

## Supplementary Materials

The following supporting information can be downloaded at: https://www.mdpi.com/article/10.3390/genes14010185/s1, Figure S1.1: Age and acceleration value of biological age calculated by the PhenoAge model in the study group of individuals depending on the genotypes of the polymorphism *rs5742904* of the *ApoB* gene; Figure S1.2: Indicators of systolic (a) and diastolic (b) blood pressure (BP) in the study group of individuals, depending on the genotypes of polymorphism *rs5742904* of the *ApoB* gene; Figure S1.3: Value of carotid-femoral pulse wave velocity (cfPWV) in the study group of individuals, depending on the genotypes of polymorphism *rs5742904* of the *ApoB* gene; Figure S1.4: Boxplot of ABI right and left limbs for APOB groups; Figure S1.5: Body mass index (BMI) values in the study group of individuals depending on the genotypes of polymorphism *rs5742904* of the *ApoB* gene; Figure S1.6: Boxplot of Pulse pressure and Pulse rate for APOB groups; Figure S1.7: Number of subjects (count of participants) depends on APOB for Arrhythmia and for Hypertension absence/presence; Figure S2.1: Age andacceleration valueofbiological age calculated by the PhenoAge model in the study groupofindividuals depending on the genotypes ofthe polymorphism *rs1107946* of the *COLlAl* gene; Figure S2.2: Indicators ofsystolic (a) and diastolic (b) blood pressure (BP) in the study group of individuals, depending on thegenotypes ofpolymorphism *rs1107946* of the *COLlAl* gene; Figure S2.3: Valueofcarotid-femoral pulse wave velocity(cfPWV) in the study group of individuals depending on the genotypes of polymorphism *rs1107946* of the *COLlAl* gene; Figure S2.4: Boxplot of ABI right and left limbs for *COLlAl* groups; Figure S2.5:Bodymassindex(BM)values in the study group ofindividuals depending on the genotypes of polymorphism *rs1107946* of the *COLlAl* gene; Figure S2.6: Boxplot of Pulse pressure and Pulse rate for COLlAl groups; Figure S2.7: Number ofsubjects(count ofparticipants) depends on *COLlAl* for Arrhythmia and for Hypertension absence/presence; Figure S3.1: Ageandaccelerationvalueofbiological age calculated by the PhenoAge model in the stud groupofindividuals depending on the genotypes of the polymorphism *rs652438* of the *MMP12* gene; Figure S3.2: Indicators ofsystolic (a) and diastolic (b) blood pressure (BP) in the study group of individuals, depending on the genotypes of polymorphism *rs652438* of the *MMP12* gene; Figure S3.3: Value ofcarotid-femoral pulse wave velocity(cfPWV) in the study group of individuals, depending on the genotypes of polymorphism *rs652438* of the *MMP12* gene; Figure S3.4: Boxplot of ABI right and left limbs for *MMP12* groups; Figure S3.5: Bodymassindex(BMI)values in the study group ofindividuals depending on the genotypes of polymorphism *rs652438* of the *MMP12* gene; Figure S3.6: Boxplot of Pulse pressure and Pulse rate for *MMP12* groups; Figure 3.7: Number ofsubjects(count of participants) depends on *MMP12* for Arrhythmia and for Hypertension absence/presence; Figure S4.1: Ageandacceleration value ofbiological age calculated by the PhenoAge model in the study groupofindividuals dependingon the genotypes ofthe polymorphism *rs1801131* of the *MTHFR* gene; Figure S4.2: Indicators of systolic (a) and diastolic (b) blood pressure (BP) in the study group of individuals, depending on the genotypes ofpolymorphism *rs1801131* of the *MTHFR* gene; Figure S4.3: Value ofcarotid-femoral pulse wave velocity(cfPWV) in the study group of individuals. depending onthe genotypes of polymorphism *rs1801131* of the *MTHFR* gene; Figure S4.4: Boxplot of ABI right and left limbs for *MTHFR* groups; Figure S4.5: Bodymassindex(BM)values in the study group ofindividuals depending on the genotypes of polymorphism *rs1801131* of the *MTHFR* gene; Figure S4.6: Boxplot of Pulse pressure and Pulse rate for *MTHFR* groups; Figure S4.7: Number ofsubjects(count of participants) depends on *MTHFR* for Arrhythmia and for Hypertension absence/presence; Figure S5.1: Scatterplot of (a) systolic bloodpressure (b) diastolic blood pressure and (c) age acceleration depends on age in different groups of polymorphic variant of the *MMP9* gene. Each point on the graph corresponds to a study participant; Figure S5.2: Histograms of age distribution depending on the presence/absence of hypertension and polymorphic variant of the *MMP9* gene. Green indicates the control- the absence of hypertension. Red. the presence of hypertension. The conditional boundary of division into groups of young and old is 60 years; Figure S5.3: The presence of hypertension in the study group of individuals depending on the sex and genotypes of the polymorphism *rs17576* ofthe *MMP9* gene; differences were assessed using a corrected *p*-value (*p*) < 0.05 (x^2^ test, post-hoc pairwise x^2^ test Benjamini-Hochberg FDR correction); Figure S6.1: Value ofage in the study group of individuals depending on the genotypes of the polymorphism *rs17576* of the *MMP9* gene; Figure S6.2: Valueofcarotid-femoral pulse wave velocity (cfPWV) in the study group of individuals. depending on the genotypes of polymorphism *rs17576* of the *MMP9* gene; Figure S6.3: Boxplot of ABI right and left limbs for *MMP9* groups; Figure S6.4: Boxplot of Pulse pressure and Pulse rate for *MMP9* groups; Figure S6.5: Number of subjects(count of participants) depends on MMP9 for Arrhythmia absence/presence.
