# Supplementary material for: New Genetically Determined Markers of the Functional State of the Cardiovascular System
Source: Genes (Basel). 2023 Jan 10;14(1):185. doi: 10.3390/genes14010185 (PMC9858790; doi:10.3390/genes14010185)
Supplement: Supplementary file 1 [file genes-14-00185-s001.zip › genes-2080711-supplementary.pdf]

Supplementary Materials for

New Genetically Determined Markers of the Functional State of the Cardiovascular System

Elena V. Kondakova [1,]*, Valeria M. Ilina [1], Lyubov M. Ermakova [1], Mikhail I. Krivonosov [1], Kirill V. Kuchin [2] and Maria V. Vedunova [1]

*Corresponding: elen_kondakova@list.ru

This section includes:

Figure S1.1 — Age and acceleration value of biological age calculated by the PhenoAge model in the study group of individuals depending on the genotypes of the polymorphism *rs5742904* of the *ApoB* gene.

Figure S1.2 — Indicators of systolic (a) and diastolic (b) blood pressure (BP) in the study group of individuals, depending on the genotypes of polymorphism *rs5742904* of the *ApoB* gene.

Figure S1.3 — Value of carotid-femoral pulse wave velocity (cfPWV) in the study group of individuals, depending on the genotypes of polymorphism *rs5742904* of the *ApoB* gene.

Figure S1.4 — Boxplot of ABI right and left limbs for APOB groups.

Figure S1.5 — Body mass index (BMI) values in the study group of individuals depending on the genotypes of polymorphism *rs5742904* of the *ApoB* gene.

Figure S1.6 — Boxplot of Pulse pressure and Pulse rate for APOB groups.

Figure S1.7— Number of subjects (count of participants) depends on APOB for Arrhythmia and for Hypertension absence/presence.

Figure S2.1 — Age and acceleration value of biological age calculated by the PhenoAge model in the study group of individuals depending on the genotypes of the polymorphism *rs1107946* of the *COL1A1* gene.

Figure S2.2 — Indicators of systolic (a) and diastolic (b) blood pressure (BP) in the study group of individuals, depending on the genotypes of polymorphism *rs1107946* of the *COL1A1* gene.

Figure S2.3 — Value of carotid-femoral pulse wave velocity (cfPWV) in the study group of individuals, depending on the genotypes of polymorphism *rs1107946* of the *COL1A1* gene.

Figure S2.4 — Boxplot of ABI right and left limbs for *COL1A1*groups.

Figure S2.5 — Body mass index (BMI) values in the study group of individuals depending on the genotypes of polymorphism *rs1107946* of the *COL1A1* gene

Figure S2.6 — Boxplot of Pulse pressure and Pulse rate for *COL1A1* groups.

Figure S2.7— Number of subjects (count of participants) depends on *COL1A1* for Arrhythmia and for Hypertension absence/presence.

Figure S3.1 — Age and acceleration value of biological age calculated by the PhenoAge model in the study group of individuals depending on the genotypes of the polymorphism *rs652438* of the *MMP12* gene.

Figure S3.2 — Indicators of systolic (a) and diastolic (b) blood pressure (BP) in the study group of individuals, depending on the genotypes of polymorphism *rs652438* of the *MMP12* gene.

Figure S3.3 — Value of carotid-femoral pulse wave velocity (cfPWV) in the study group of individuals, depending on the genotypes of polymorphism *rs652438* of the *MMP12* gene.

Figure S3.4 — Boxplot of ABI right and left limbs for *MMP12* groups.

Figure S3.5 — Body mass index (BMI) values in the study group of individuals depending on the genotypes of polymorphism *rs652438* of the *MMP12* gene.

Figure S3.6 — Boxplot of Pulse pressure and Pulse rate for *MMP12* groups.

Figure S3.7— Number of subjects (count of participants) depends on *MMP12* for Arrhythmia and for Hypertension absence/presence.

Figure S4.1 — Age and acceleration value of biological age calculated by the PhenoAge model in the study group of individuals depending on the genotypes of the polymorphism *rs1801131* of the *MTHFR* gene.

Figure S4.2 — Indicators of systolic (a) and diastolic (b) blood pressure (BP) in the study group of individuals, depending on the genotypes of polymorphism *rs1801131* of the *MTHFR* gene.

Figure S4.3 — Value of carotid-femoral pulse wave velocity (cfPWV) in the study group of individuals, depending on the genotypes of polymorphism *rs1801131* of the *MTHFR* gene.

Figure S4.4 — Boxplot of ABI right and left limbs for *MTHFR* groups.

Figure S4.5 — Body mass index (BMI) values in the study group of individuals depending on the genotypes of polymorphism *rs1801131* of the *MTHFR* gene.

Figure S4.6 — Boxplot of Pulse pressure and Pulse rate for *MTHFR* groups.

Figure S4.7— Number of subjects (count of participants) depends on *MTHFR* for Arrhythmia and for Hypertension absence/presence.

Figure S5.1 — Scatterplot of (a) systolic blood pressure (b) diastolic blood pressure and (c) age acceleration depends on age in different groups of polymorphic variant of the *MMP9* gene. Each point on the graph corresponds to a study participant.

Figure S5.2 — Histograms of age distribution depending on the presence/absence of hypertension and polymorphic variant of the *MMP9* gene. Green indicates the control - the absence of hypertension. Red - the presence of hypertension. The conditional boundary of division into groups of young and old is 60 years.

Figure S5.3 — The presence of hypertension in the study group of individuals depending on the sex and genotypes of the polymorphism *rs17576* of the *MMP9* gene; differences were assessed using a corrected p-value (p) < 0.05 ($\chi2$ test, post-hoc pairwise $\chi2$ test Benjamini–Hochberg FDR correction).

Figure S6.1 — Value of age in the study group of individuals depending on the genotypes of the polymorphism *rs17576* of the *MMP9* gene.

Figure S6.2 — Value of carotid-femoral pulse wave velocity (cfPWV) in the study group of individuals, depending on the genotypes of polymorphism *rs17576* of the *MMP9* gene.

Figure S6.3 — Boxplot of ABI right and left limbs for *MMP9* groups.

Figure S6.4 — Boxplot of Pulse pressure and Pulse rate for *MMP9* groups.

Figure S6.5— Number of subjects (count of participants) depends on MMP9 for Arrhythmia absence/presence.

Apo B

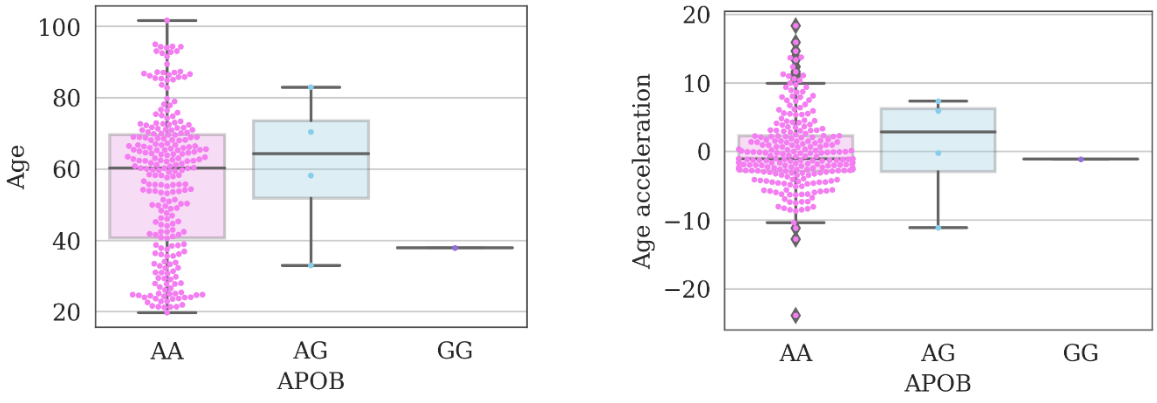

(a) **(b)**

Figure S1.1 — Age (a) and acceleration value of biological age calculated by the PhenoAge model (b) in the study group of individuals depending on the genotypes of the polymorphism *rs5742904* of the *ApoB* gene. No significant differences at a corrected p-value (p) < 0.05 (Kruskal–Wallis test).

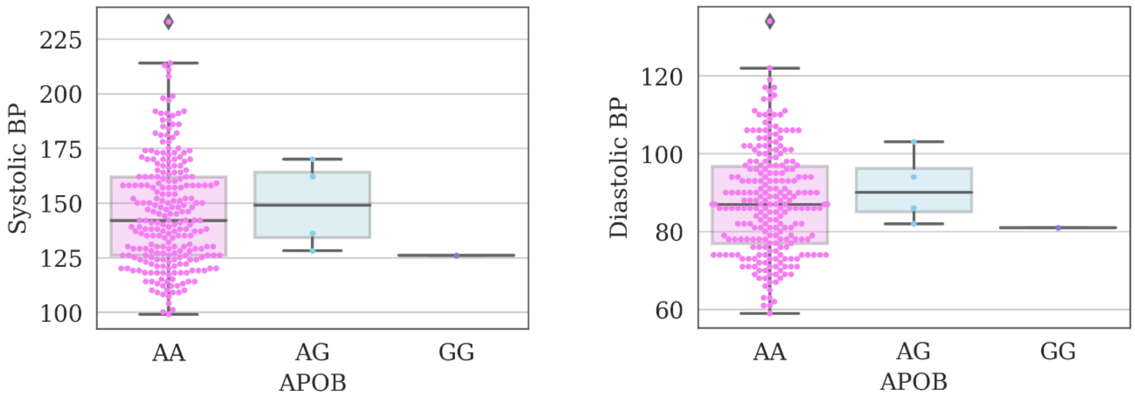

(a) **(b)**

Figure S1.2 — Indicators of systolic (a) and diastolic (b) blood pressure (BP) in the study group of individuals, depending on the genotypes of polymorphism *rs5742904* of the *ApoB* gene. No significant differences at a corrected p-value (p) < 0.05 (Kruskal–Wallis test).

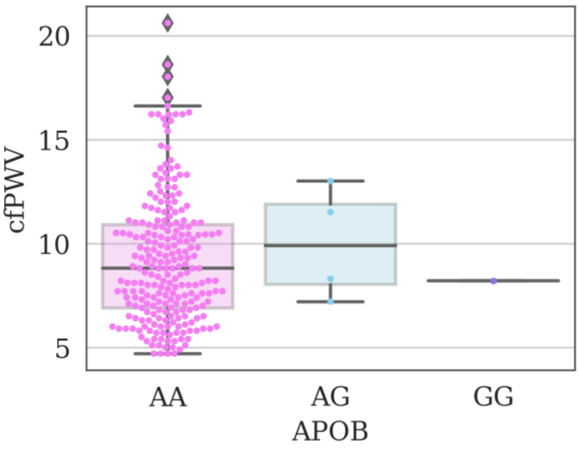

Figure S1.3 — Value of carotid-femoral pulse wave velocity (cfPWV) in the study group of individuals, depending on the genotypes of polymorphism *rs5742904* of the *ApoB* gene. No significant differences at a corrected p-value (p) < 0.05 (Kruskal–Wallis test).

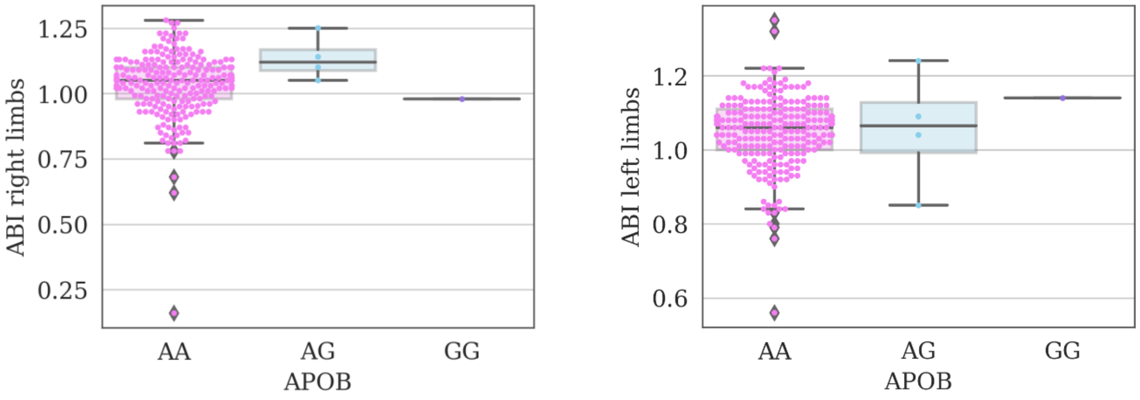

Figure S1.4 — Boxplot of ABI right and left limbs for *APOB* groups. No significant differences at a corrected p-value (p) < 0.05 (Kruskal–Wallis test).

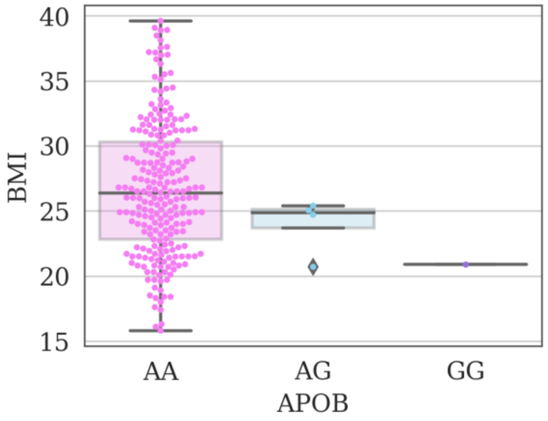

Figure S1.5 — Body mass index (BMI) values in the study group of individuals depending on the genotypes of polymorphism *rs5742904* of the *ApoB* gene. No significant differences at a corrected p-value (p) < 0.05 (Kruskal–Wallis test).

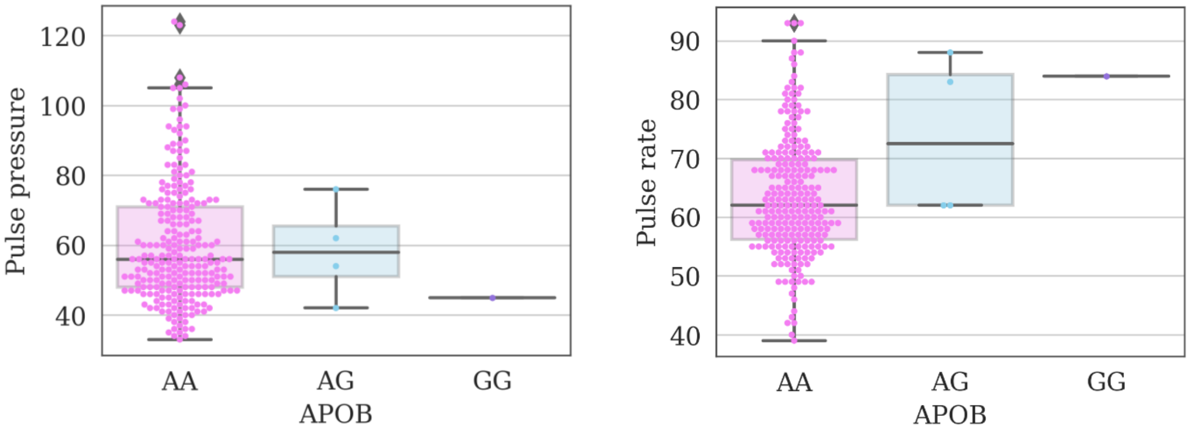

Figure S1.6 — Boxplot of Pulse pressure and Pulse rate for *APOB* groups. No significant differences at a corrected p-value (p) < 0.05 (Kruskal–Wallis test).

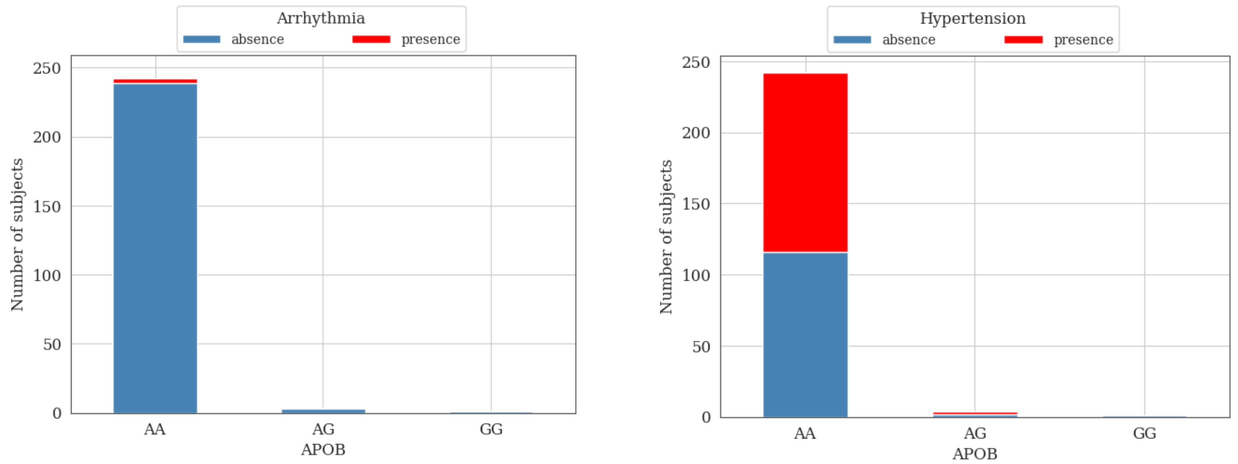

Figure S1.7— Number of subjects (count of participants) depends on *APOB* for Arrhythmia and for Hypertension absence/presence. No significant differences at a corrected p-value (p) < 0.05 (χ2 test).

COL1A1

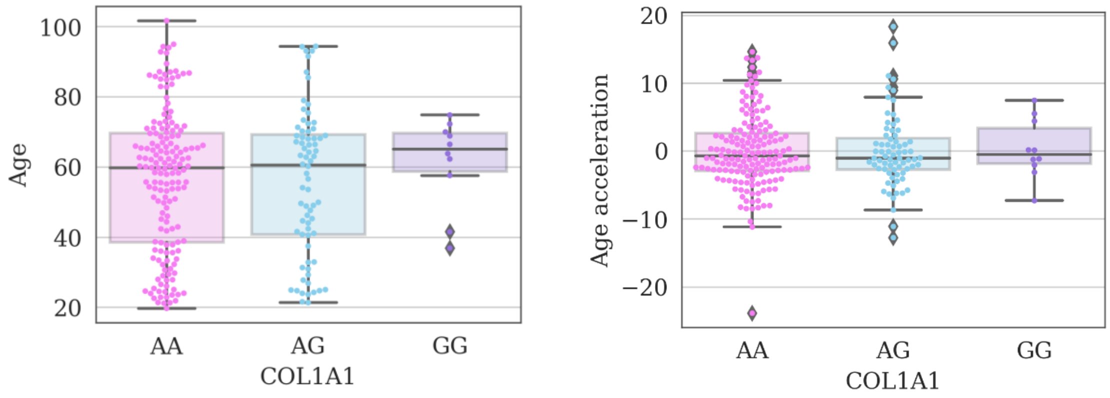

(a) (b)

Figure S2.1 — Age (a) and acceleration value of biological age calculated by the PhenoAge model (b) in the study group of individuals depending on the genotypes of the polymorphism *rs1107946* of the *COL1A1* gene. No significant differences at a corrected p-value (p) < 0.05 (Kruskal–Wallis test).

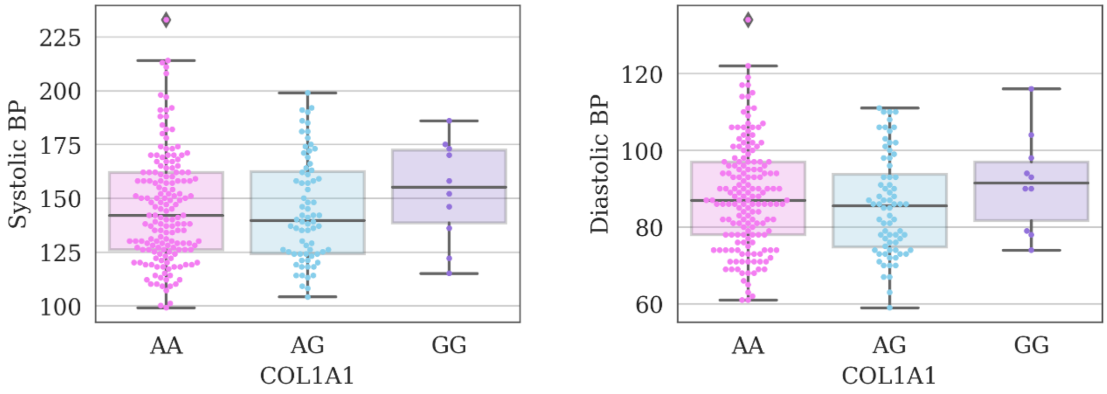

(a) (b)

Figure S2.2 — Indicators of systolic (a) and diastolic (b) blood pressure (BP) in the study group of individuals, depending on the genotypes of polymorphism *rs1107946* of the *COL1A1* gene. No significant differences at a corrected p-value (p) < 0.05 (Kruskal–Wallis test).

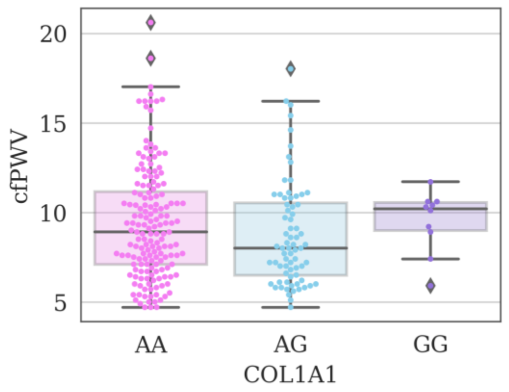

Figure S2.3 — Value of carotid-femoral pulse wave velocity (cfPWV) in the study group of individuals, depending on the genotypes of polymorphism *rs1107946* of the *COL1A1* gene. No significant differences at a corrected p-value (p) < 0.05 (Kruskal–Wallis test).

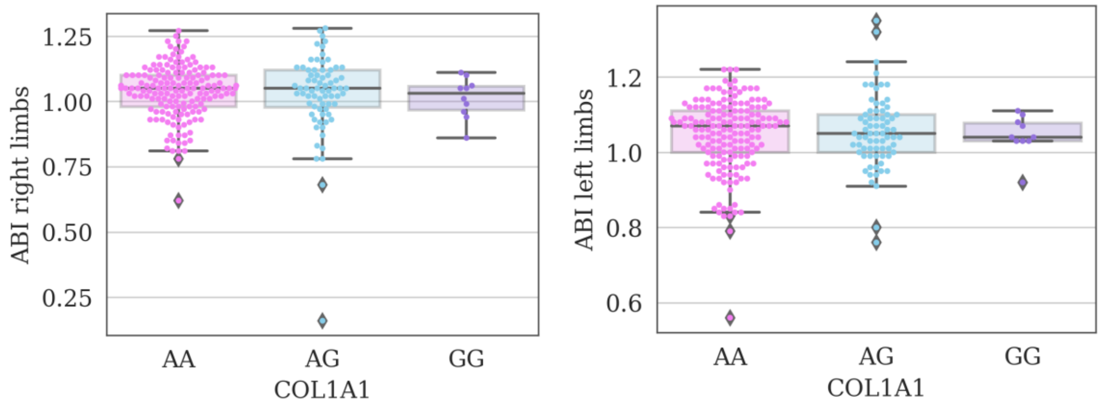

Figure S2.4 — Boxplot of ABI right and left limbs for *COL1A1* groups. No significant differences at a corrected p-value (p) < 0.05 (Kruskal–Wallis test).

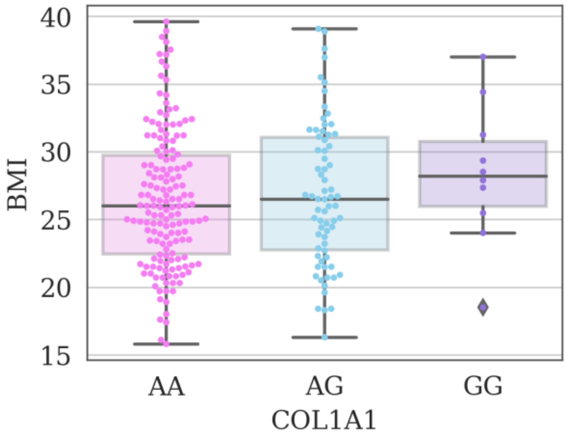

Figure S2.5 — Body mass index (BMI) values in the study group of individuals depending on the genotypes of polymorphism *rs1107946* of the *COL1A1* gene. No significant differences at a corrected p-value (p) < 0.05 (Kruskal–Wallis test).

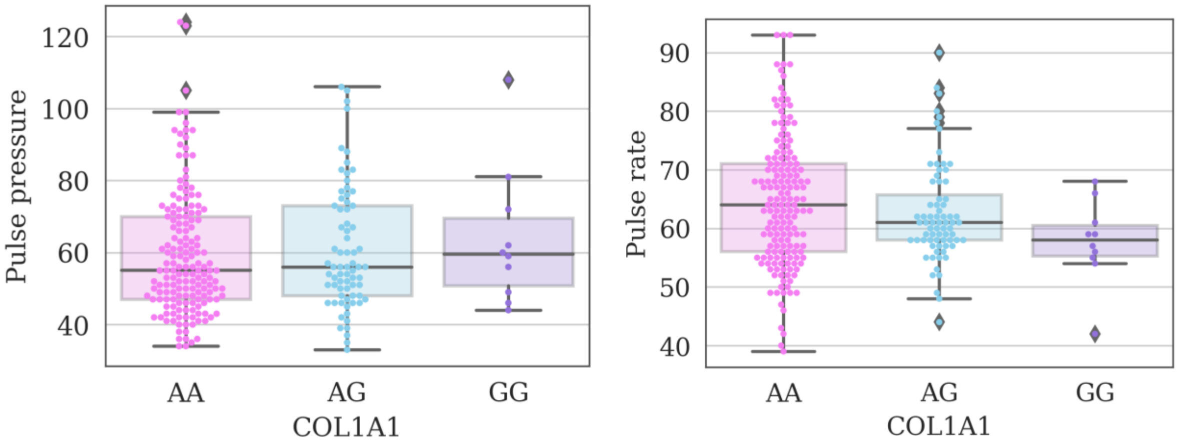

Figure S2.6 — Boxplot of Pulse pressure and Pulse rate for *COL1A1* groups. No significant differences at a corrected p-value (p) < 0.05 (Kruskal–Wallis test).

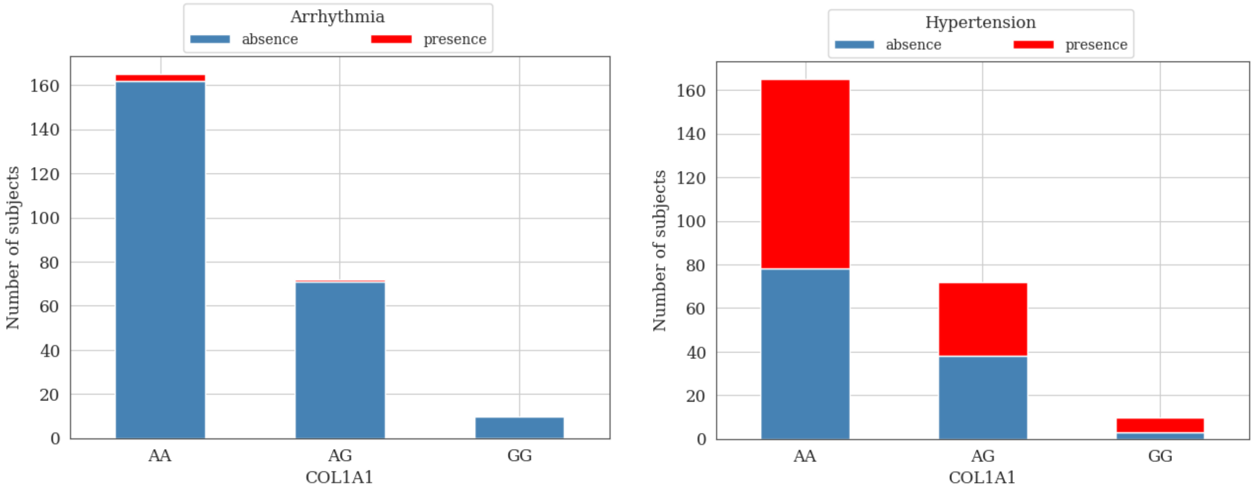

Figure S2.7— Number of subjects (count of participants) depends on *COL1A1* for Arrhythmia and for Hypertension absence/presence. No significant differences at a corrected p-value (p) < 0.05 (χ2 test).

MMP12

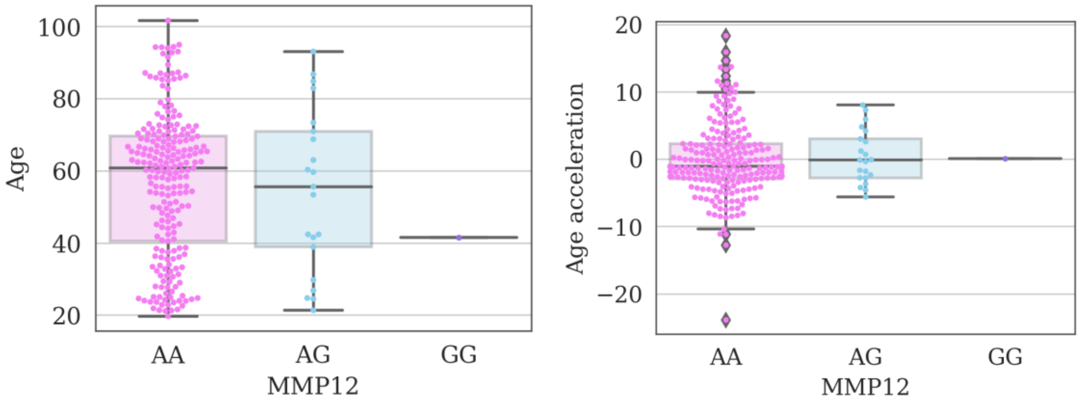

Figure S3.1 — Age and acceleration value of biological age calculated by the PhenoAge model in the study group of individuals depending on the genotypes of the polymorphism *rs652438* of the *MMP12* gene. No significant differences at a corrected p-value (p) < 0.05 (Kruskal–Wallis test).

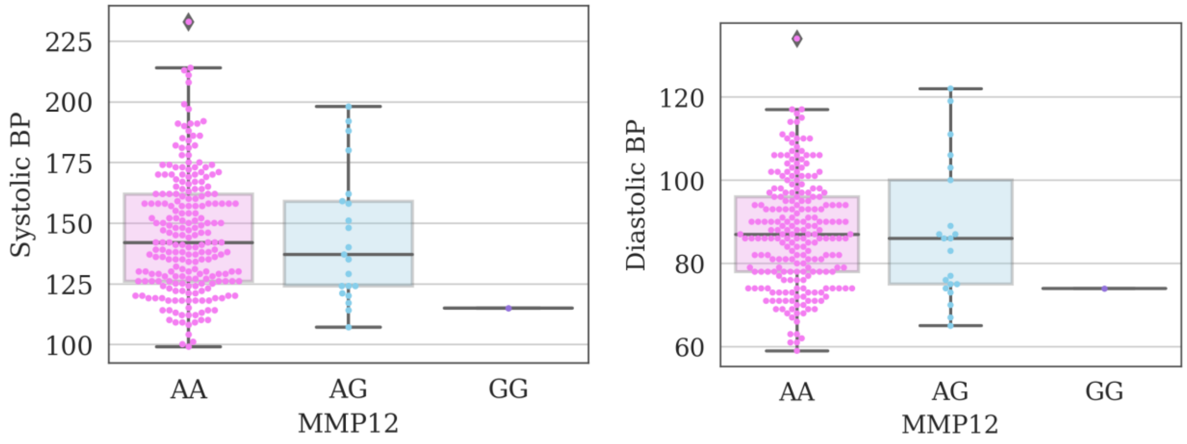

Figure S3.2 — Indicators of systolic (a) and diastolic (b) blood pressure (BP) in the study group of individuals, depending on the genotypes of polymorphism *rs652438* of the *MMP12* gene. No significant differences at a corrected p-value (p) < 0.05 (Kruskal–Wallis test).

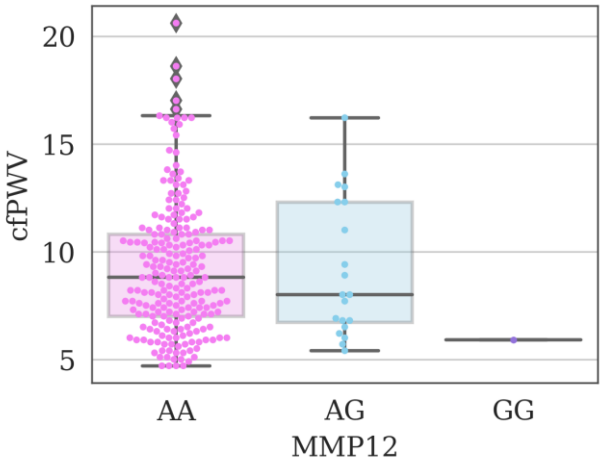

Figure S3.3 — Value of carotid-femoral pulse wave velocity (cfPWV) in the study group of individuals, depending on the genotypes of polymorphism *rs652438* of the *MMP12* gene. No significant differences at a corrected p-value (p) < 0.05 (Kruskal–Wallis test).

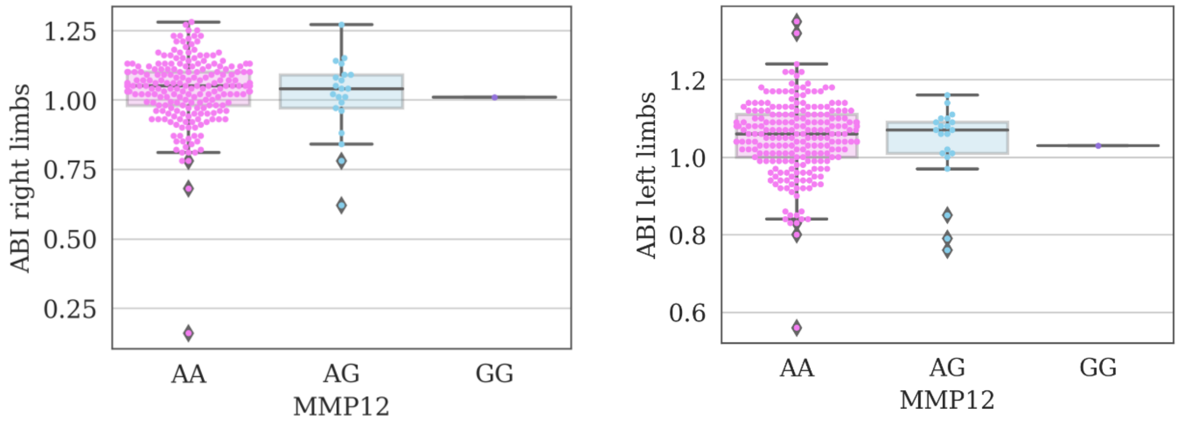

Figure S3.4 — Boxplot of ABI right and left limbs for *MMP12* groups. No significant differences at a corrected p-value (p) < 0.05 (Kruskal–Wallis test).

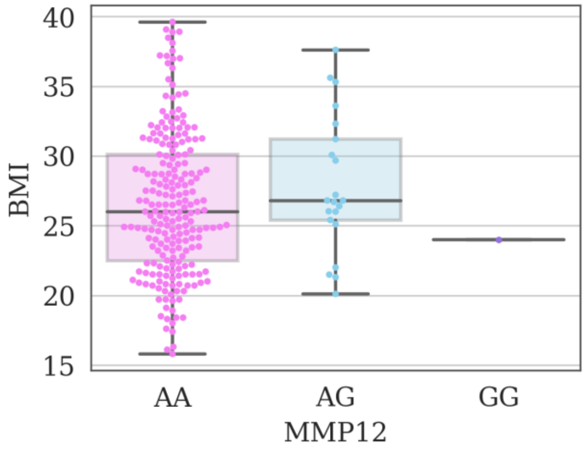

Figure S3.5 — Body mass index (BMI) values in the study group of individuals depending on the genotypes of polymorphism *rs652438* of the *MMP12* gene. No significant differences at a corrected p-value (p) < 0.05 (Kruskal–Wallis test).

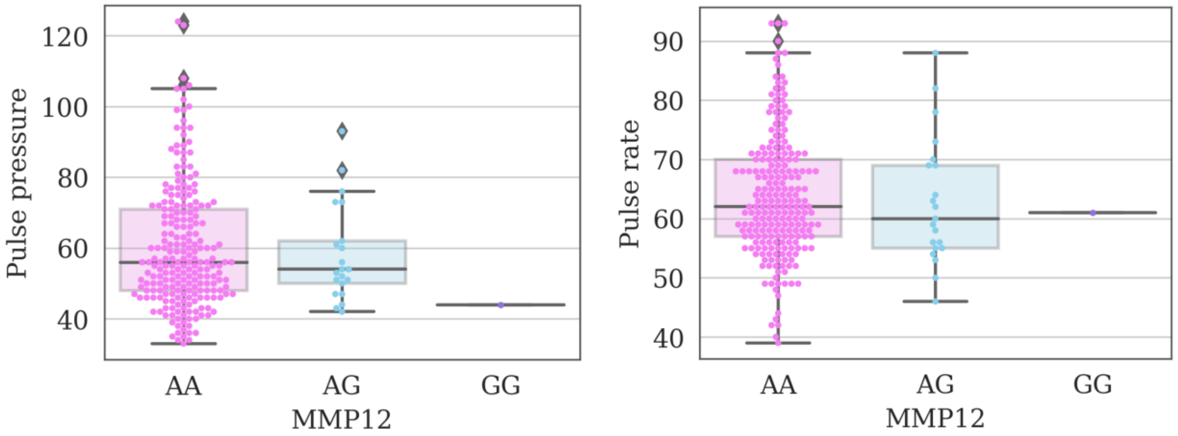

Figure S3.6 — Boxplot of Pulse pressure and Pulse rate for *MMP12* groups. No significant differences at a corrected p-value (p) < 0.05 (Kruskal–Wallis test).

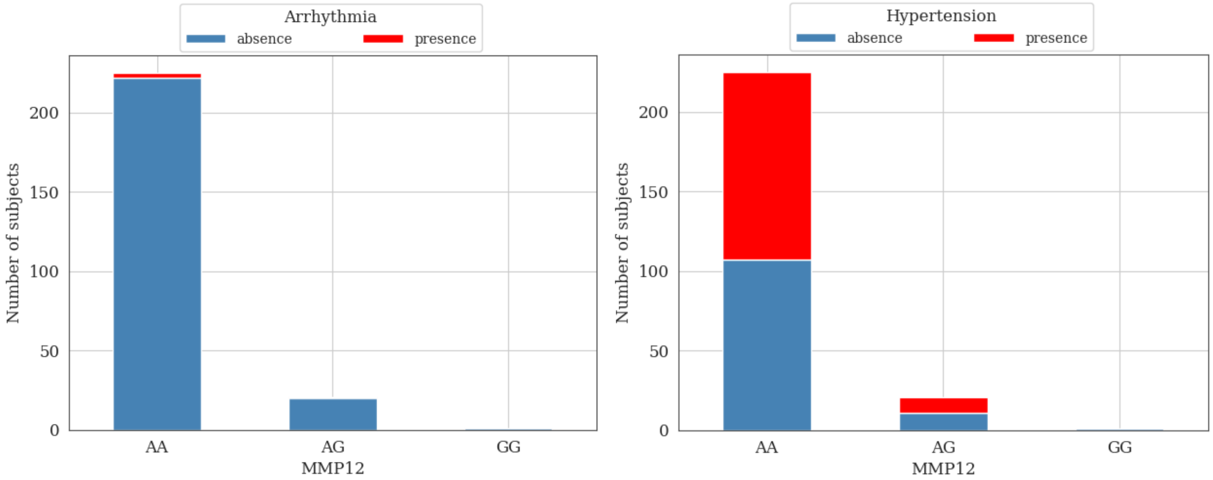

Figure S3.7— Number of subjects (count of participants) depends on *MMP12* for Arrhythmia and for Hypertension absence/presence. No significant differences at a corrected p-value (p) < 0.05 (χ2 test).

MTHFR

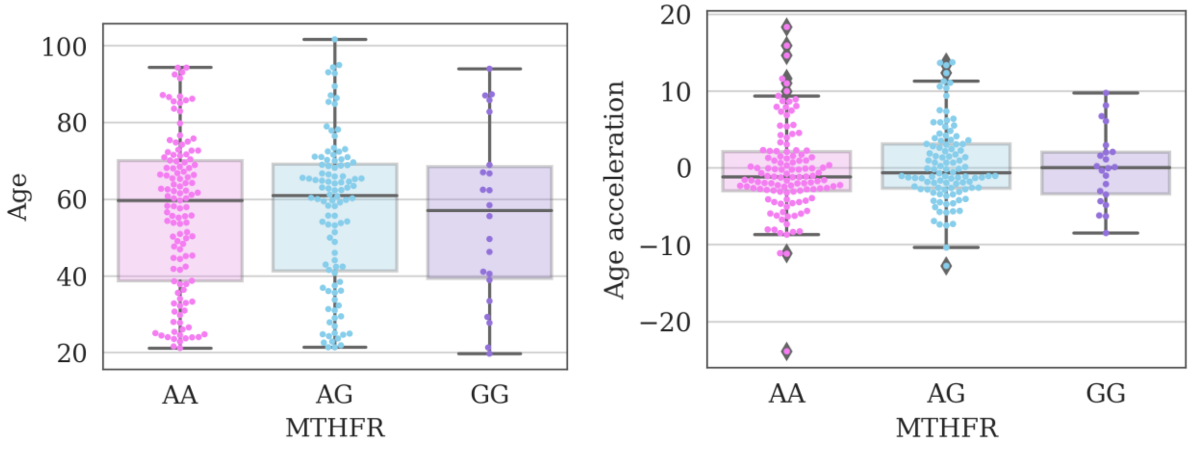

Figure S4.1 — Age and acceleration value of biological age calculated by the PhenoAge model in the study group of individuals depending on the genotypes of the polymorphism *rs1801131* of the *MTHFR* gene. No significant differences at a corrected p-value (p) < 0.05 (Kruskal–Wallis test).

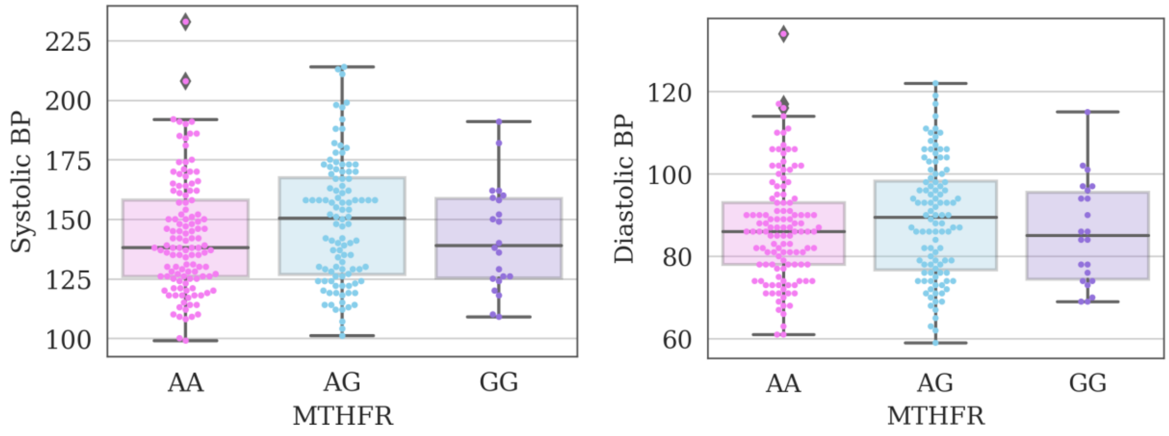

Figure S4.2 — Indicators of systolic (a) and diastolic (b) blood pressure (BP) in the study group of individuals, depending on the genotypes of polymorphism *rs1801131* of the *MTHFR* gene. No significant differences at a corrected p-value (p) < 0.05 (Kruskal–Wallis test).

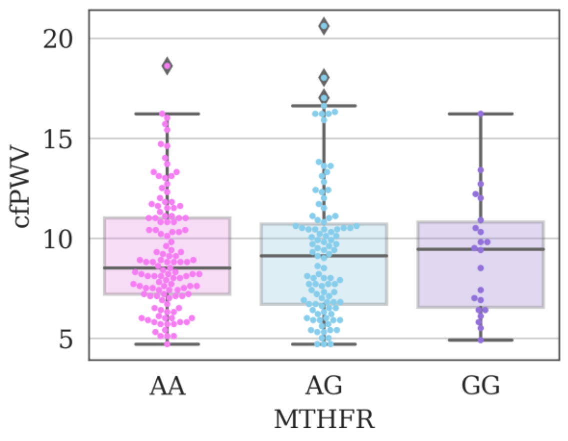

Figure S4.3 — Value of carotid-femoral pulse wave velocity (cfPWV) in the study group of individuals, depending on the genotypes of polymorphism *rs1801131* of the *MTHFR* gene. No significant differences at a corrected p-value (p) < 0.05 (Kruskal–Wallis test).

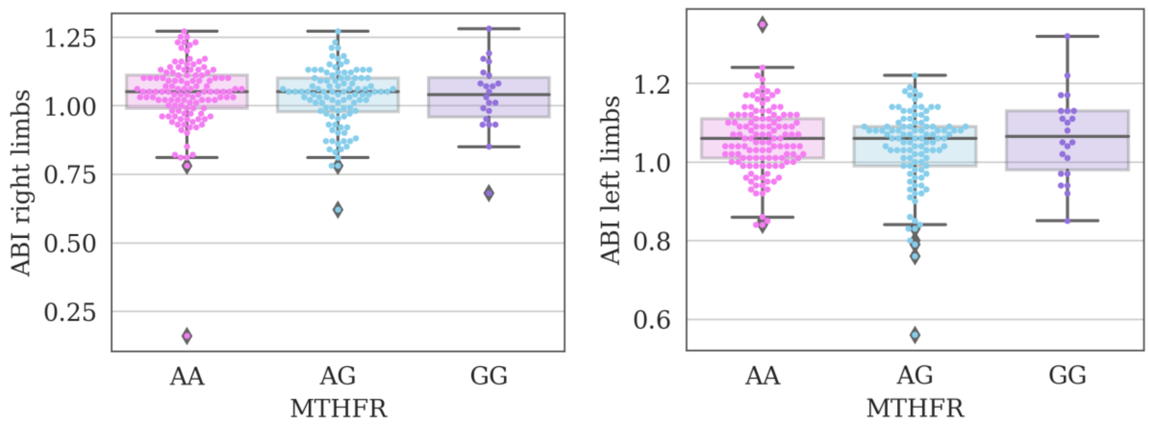

Figure S4.4 — Boxplot of ABI right and left limbs for *MTHFR* groups. No significant differences at a corrected p-value (p) < 0.05 (Kruskal–Wallis test).

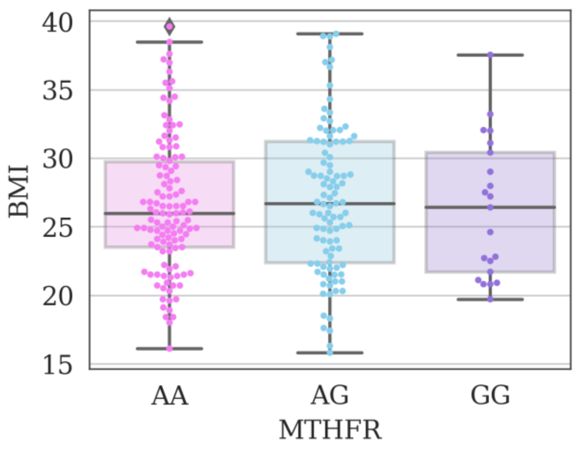

Figure S4.5 — Body mass index (BMI) values in the study group of individuals depending on the genotypes of polymorphism *rs1801131* of the *MTHFR* gene. No significant differences at a corrected p-value (p) < 0.05 (Kruskal–Wallis test).

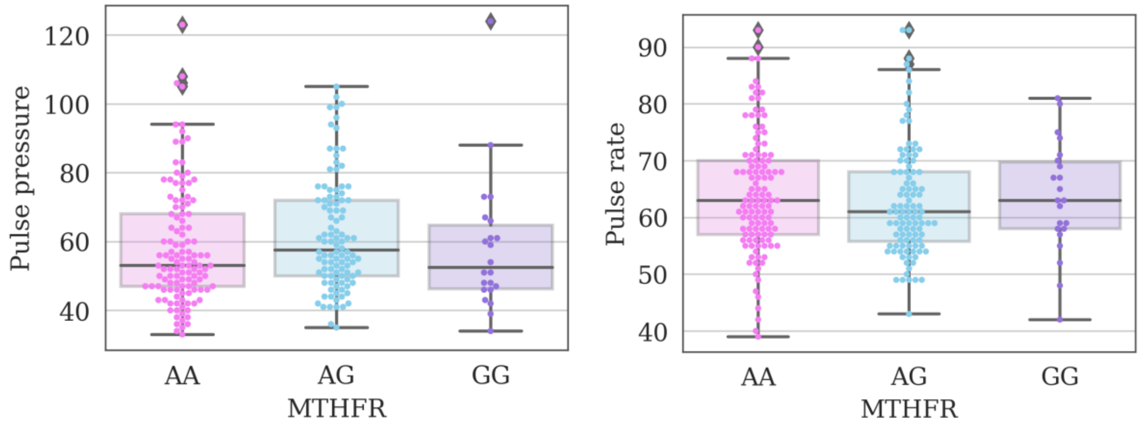

Figure S4.6 — Boxplot of Pulse pressure and Pulse rate for *MTHFR* groups. No significant differences at a corrected p-value (p) < 0.05 (Kruskal–Wallis test).

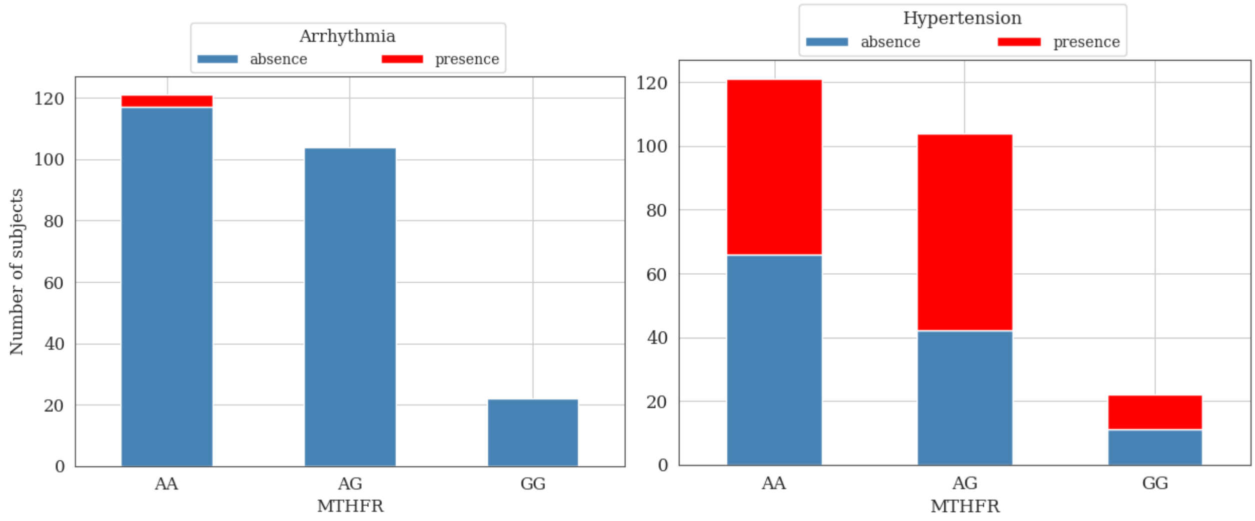

Figure S4.7— Number of subjects (count of participants) depends on *MTHFR* for Arrhythmia and for Hypertension absence/presence. No significant differences at a corrected p-value (p) < 0.05 ($\chi 2$ test).

MMP9

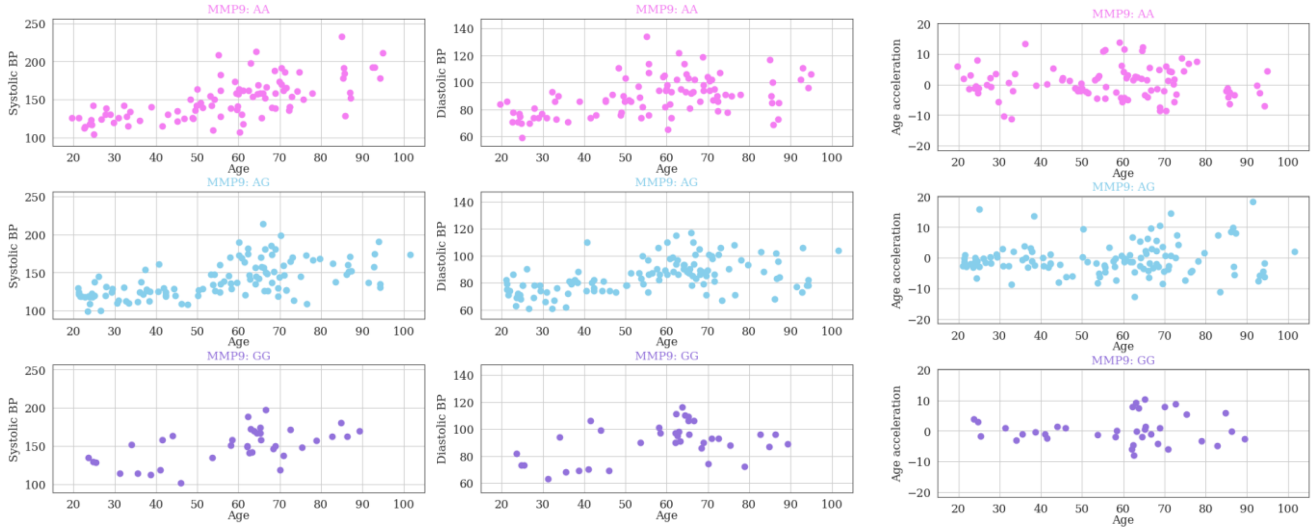

(a) (b) (c)

Figure S5.1 — Scatterplot of (a) systolic blood pressure (b) diastolic blood pressure and (c) age acceleration depends on age in different groups of polymorphic variant of the *MMP9* gene. Each point on the graph corresponds to a study participant.

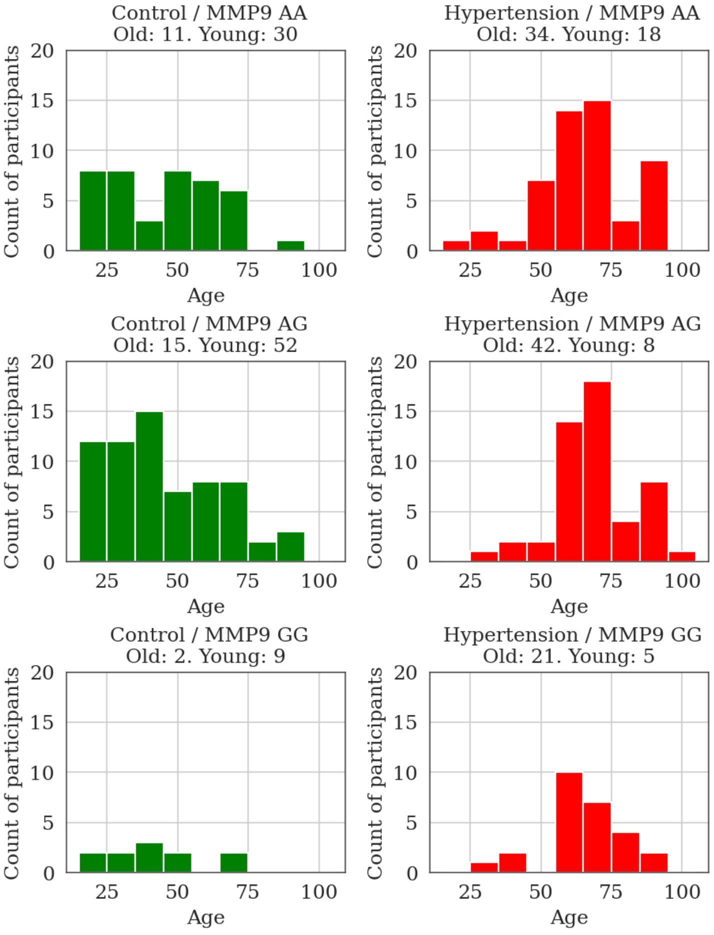

Figure S5.2 — Histograms of age distribution depending on the presence/absence of hypertension and polymorphic variant of the *MMP9* gene. Green indicates the control - the absence of hypertension. Red - the presence of hypertension. The conditional boundary of division into groups of young and old is 60 years.

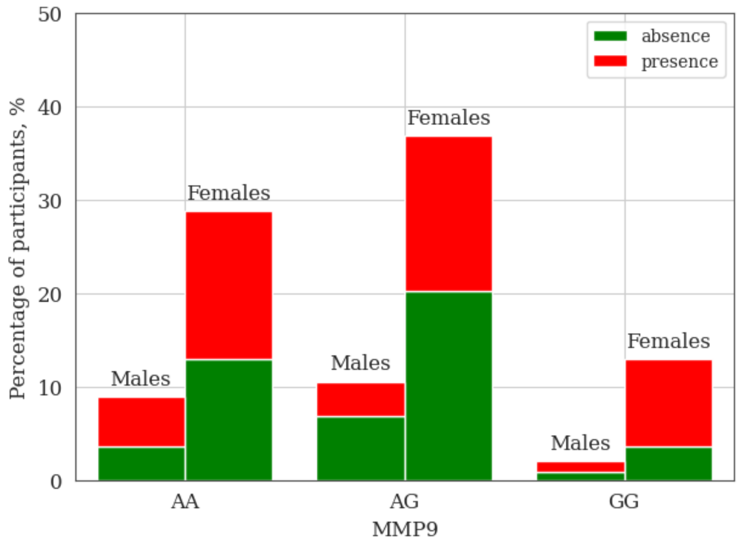

Figure S5.3 — The presence of hypertension in the study group of individuals depending on the sex and genotypes of the polymorphism *rs17576* of the *MMP9* gene; differences were assessed using a corrected p-value (p) < 0.05 (χ^2 test, post-hoc pairwise χ^2 test Benjamini–Hochberg FDR correction).

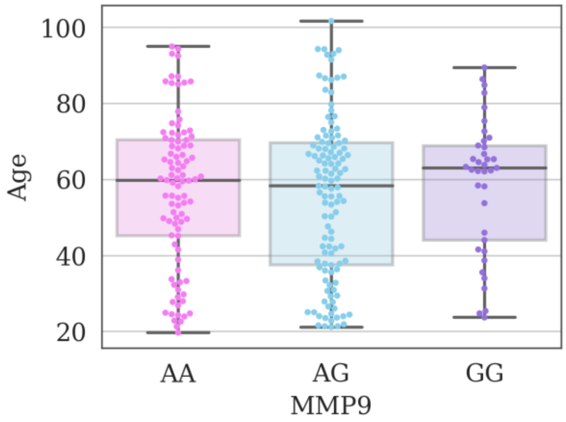

Figure S6.1 — Value of age in the study group of individuals depending on the genotypes of the polymorphism *rs17576* of the *MMP9* gene. No significant differences at a corrected p-value (p) < 0.05 (Kruskal–Wallis test).

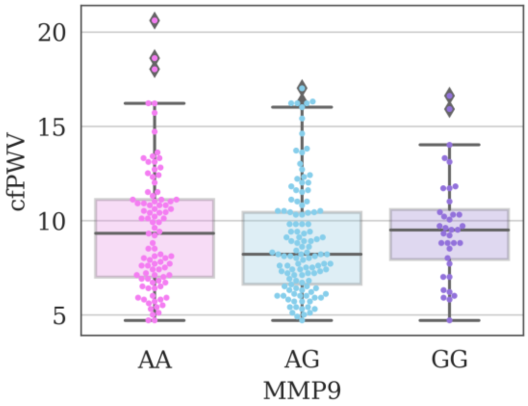

Figure S6.2 — Value of carotid-femoral pulse wave velocity (cfPWV) in the study group of individuals, depending on the genotypes of polymorphism *rs17576* of the *MMP9* gene. No significant differences at a corrected p-value (p) < 0.05 (Kruskal–Wallis test).

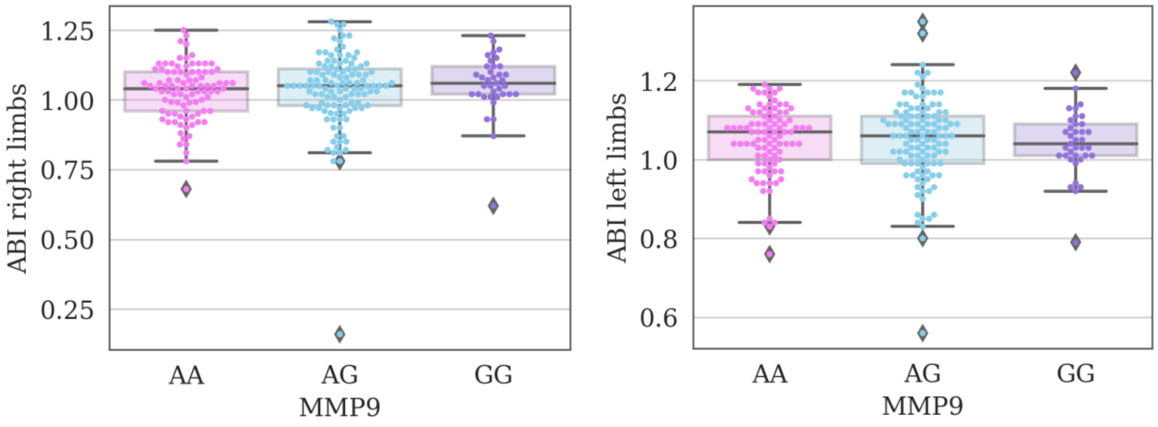

Figure S6.3 — Boxplot of ABI right and left limbs for *MMP9* groups. No significant differences at a corrected p-value (p) < 0.05 (Kruskal–Wallis test).

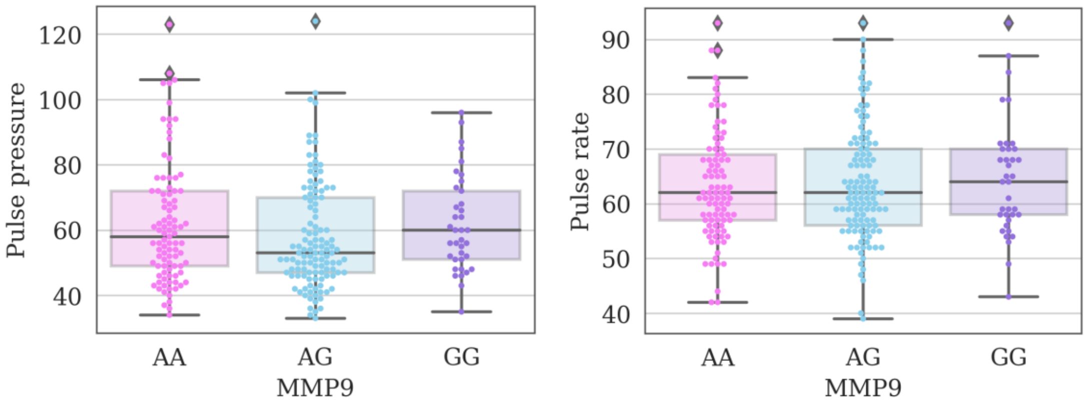

Figure S6.4 — Boxplot of Pulse pressure and Pulse rate for *MMP9* groups. No significant differences at a corrected p-value (p) < 0.05 (Kruskal–Wallis test).

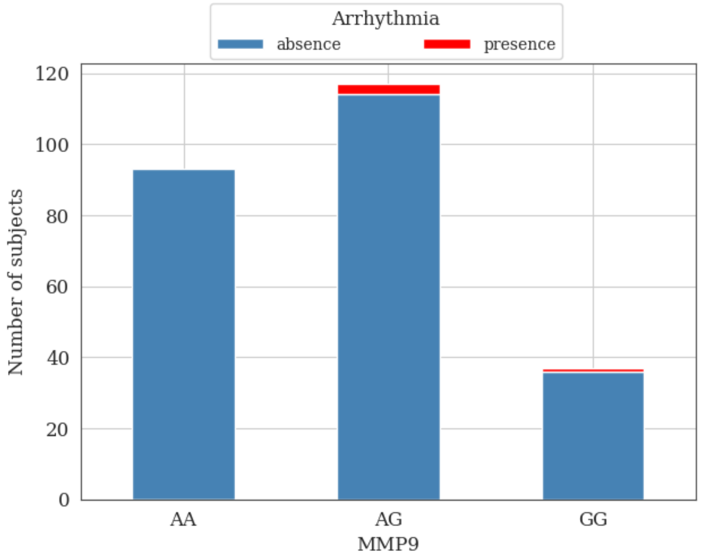

Figure S6.5— Number of subjects (count of participants) depends on *MMP9* for Arrhythmia absence/presence. No significant differences at a corrected p-value (p) < 0.05 ($\chi 2$ test).
